# Substantial Morbidity and Mortality Associated with Pandemic A/H1N1 Influenza in Mexico, Winter 2013-2014: Gradual Age Shift and Severity

**DOI:** 10.1371/currents.outbreaks.a855a92f19db1d90ca955f5e908d6631

**Published:** 2014-03-26

**Authors:** Javier Dávila, Gerardo Chowell, Víctor H. Borja-Aburto, Cécile Viboud, Concepciòn Grajales Muñiz, Mark Miller

**Affiliations:** Dirección de Prestaciones Médicas, Instituto Mexicano del Seguro Social, Mexico City, México; Division of Epidemiology and Population Studies, Fogarty International Center, National Institutes of Health, Bethesda, Maryland, USA; Mathematical, Computational & Modeling Sciences Center, School of Human Evolution and Social Change, Arizona State University, Tempe, Arizona, USA; Dirección de Prestaciones Médicas, Instituto Mexicano del Seguro Social, Mexico City, México; Division of Epidemiology and Population Studies, Fogarty International Center, National Institutes of Health, Bethesda, Maryland, USA; Coordinación de Vigilancia Epidemiológica y Apoyo en Contingencias, Instituto Mexicano del Seguro Social, Mier y Pesado 120, México, DF 03100 México; Division of Epidemiology and Population Studies, Fogarty International Center, National Institutes of Health, Bethesda, Maryland, USA

**Keywords:** A/H1N1, Influenza, Mexico, Pandemic

## Abstract

Background: A recrudescent wave of pandemic influenza A/H1N1 is underway in Mexico in winter 2013-14, following a mild 2012-13 A/H3N2 influenza season. Mexico previously experienced several waves of pandemic A/H1N1 activity in spring, summer and fall 2009 and winter 2011-2012, with a gradual shift of influenza-related hospitalizations and deaths towards older ages. Here we describe changes in the epidemiology of the 2013-14 A/H1N1 influenza outbreak, relative to previous seasons dominated by the A/H1N1 pandemic virus. The analysis is intended to guide public health intervention strategies in near real time. Methods: We analyzed demographic and geographic data on hospitalizations with severe acute respiratory infection (SARI), laboratory-confirmed A/H1N1 influenza hospitalizations, and inpatient deaths, from a large prospective surveillance system maintained by the Mexican Social Security medical system during 01-October 2013 to 31-Jan 2014. We characterized the age and regional patterns of influenza activity relative to the preceding 2011-2012 A/H1N1 influenza epidemic. We also estimated the reproduction number (R) based on the growth rate of daily case incidence by date of symptoms onset. Results: A total of 7,886 SARI hospitalizations and 529 inpatient-deaths (3.2%) were reported between 01-October 2013 and 31-January 2014 (resulting in 3.2 laboratory-confirmed A/H1N1 hospitalizations per 100,00 and 0.52 laboratory-confirmed A/H1N1-positive deaths per 100,000). The progression of daily SARI hospitalizations in 2013-14 exceeded that observed during the 2011-2012 A/H1N1 epidemic. The mean age of laboratory-confirmed A/H1N1 patients in 2013-14 was 41.1 y (SD=20.3) for hospitalizations and 49.2 y (SD=16.7) for deaths. Rates of laboratory-confirmed A/H1N1 hospitalizations and deaths were significantly higher among individuals aged 30-59 y and lower among younger age groups for the ongoing 2013-2014 epidemic, compared to the 2011-12 A/H1N1 epidemic (Chi-square test, P<0.001). The reproduction number of the winter 2013-14 wave in central Mexico was estimated at 1.3-1.4 which is slightly higher than that reported for the 2011-2012 A/H1N1 epidemic. Conclusions: We have documented a substantial and ongoing increase in the number of A/H1N1-related hospitalizations and deaths during the period October 2013-January 2014 and a proportionate shift of severe disease to middle aged adults, relative to the preceding A/H1N1 2011-2012 epidemic in Mexico. In the absence of clear antigenic drift in globally circulating A/H1N1 viruses in the post-pandemic period, the gradual change in the age distribution of A/H1N1 infections observed in Mexico suggests a slow build-up of immunity among younger populations, reminiscent of the age profile of past pandemics.

## INTRODUCTION

The resurgence of swine-origin pandemic A/H1N1 influenza virus in winter 2013-14 is causing substantial morbidity and mortality in Mexico at the time of writing of this report. Mexico has experienced a series of three A/H1N1 pandemic waves in the spring, summer, and fall of 2009 1 ^,^ 2, and a recrudescent fourth wave in the winter of 2011-2012 3. Mexico experienced particularly high excess mortality associated with pandemic A/H1N1 influenza in 2009, relative to other countries 4 ^,^ 5. In contrast, the 2012-13 influenza season was mild and dominated by the A/H3N2 virus.

Here we report preliminary findings on the epidemiology of the on-going A/H1N1 outbreak in Mexico from October 2013 to January 2014. Because past influenza pandemics have had substantial morbidity and mortality burden for several seasons after the initial pandemic waves 6 7 8 9 10 , continued vigilance is prudent. We compare the demographic and clinical characteristics of laboratory-confirmed A/H1N1 hospitalizations and deaths in winter 2013-14 with those reported for the preceding 2011-12 A/H1N1 epidemic. Our data highlight a change in the age distribution of A/H1N1 patients and a slightly higher reproduction number compared to the 2011-12 A/H1N1 outbreak.

## MATERIALS AND METHODS

**Epidemiological Data**

Individual level hospitalization data were available from a prospective influenza surveillance system that was initiated especially for the 2009 pandemic by the Mexican Institute for Social Security (IMSS) 1 ^,^ 11. IMSS is a tripartite Mexican health system covering approximately 40% of the Mexican population comprising workers in the private sector and their families, relying on a network of 1,099 primary health-care units and 259 hospitals nationwide. The age and gender distributions of persons affiliated to the IMSS medical system are representative of the general Mexican population 1.

We analyzed all patients admitted to the hospital for > 24 hrs with a diagnosis of severe acute respiratory infection (SARI) during October 2013 to January 2014. SARI was defined as the presence of respiratory difficulty with fever >38°C and cough, together with one or more of the following clinical symptoms: confinement to bed, thoracic pain, polypnea, or acute respiratory distress syndrome. Children <5 years with pneumonia or severe pneumonia that required hospitalization were also considered as SARI cases. Respiratory swabs were obtained for about 52% of SARI hospitalizations (SARI) in winter 2013-14 and were tested for the influenza virus by RT-PCR^12^. For reference, the average influenza testing rate was 26% in the IMSS system for the 2011-2012 A/H1N1 epidemic 3, and 33% during the 2009 pandemic period^1^.

For all SARI hospitalizations, we retrieved age, gender, influenza laboratory test results (for patients tested), discharge status (alive or deceased), reporting state (including 31 states plus the Federal District), and self-reported onset dates of symptoms. We also obtained population data by state and age group for all persons affiliated with IMSS to calculate incidence rates.

**Age distribution and severity of A/H1N1 influenza in winter 2013-2014**

We examined the age distribution of all SARI hospitalizations, laboratory-confirmed influenza A/H1N1 hospitalizations and deaths from October 2013 to January 2014 with those associated with 2011-12 A/H1N1 epidemic from 01-October 2011 to 15-March 2012, using the same IMSS reporting system.

We also calculated preliminary estimates of in-hospital case fatality rate for SARI and laboratory-confirmed A/H1N1 influenza. These estimates are preliminary as we likely underestimate the true fatality ratio due to a delay from symptoms onset to death and laboratory confirmation.

**Spatial distribution of A/H1N1 influenza in winter 2013-14 and** reproduction number estimates****

We analyzed the geographic dissemination of the 2013-2014 A/H1N1 outbreak in Mexico based on state- and age-specific time series of laboratory-confirmed hospitalizations by day of symptom onset.

Further, we estimated the reproduction number, R, in Central Mexico where the great majority of A/H1N1 cases have been reported in 2013-14, based on a simple method previously used to characterize the 2009 A/H1N1 pandemic 1 and the 2011-12 A/H1N1 epidemic in Mexico 3. Specifically we estimated the initial epidemic growth rate by fitting an exponential function to entire ascending phase of daily A/H1N1 hospitalizations by date of symptoms onset 13. We assumed a mean generation interval of three and four days, which are within the range of mean estimates for the 2009 influenza pandemic 14 ^,^ 15 ^,^ 16 ^,^ 17. As a sensitivity analysis we also assessed small variations in the length of the ascending epidemic phase used to estimate the exponential growth rate.

This study did not require approval from a scientific committee; all individual data were kept de-identified.

Analyses were performed using SPSS 20.0 and Matlab (The Mathworks, Inc).

## RESULTS

**Overall epidemiological patterns**

Bewteen 01-October 2013 and 31-January 2014, 7,886 SARI hospitalizations were reported to the IMSS medical system, of which 1,203 were confirmed for A/H1N1 (Table 1). Daily time series for the last 3 consecutive seasons are shown in Figures 1 and 2. The influenza A/H1N1 percent positivity rate picked up around mid-November 2013 and A/H1N1 has remained the dominant influenza subtype this season (A/H1N1: 90.8%, H3N2: 3.7%. B: 2%); transmission is still ongoing at the time of writing of this report (Figure 2). Most of the recent A/H1N1 activity has been reported in the Central region of Mexico (55.0%), followed by southeastern states (6.5%), which was also the case in the 2011-12 outbreak (Figure 1, Table 1). Daily and cumulative numbers of SARI hospitalizations in winter 2013-14 are exceeding the levels that were observed during the 2011-2012 A/H1N1 influenza pandemic.

**Table 1. Characteristics of all SARI hospitalizations and laboratory-confirmed A/H1N1 influenza hospitalizations, Mexico, October 2013 through January 2014.** 

Variable	SARI hospitalizations (%)	A/H1N1 confirmed hospitalizations (%)
**Geographic** Central Southern Other states	3134 (39.7) 978 (12.4) 3774 (47.9)	662 (55.0) 78 (6.5) 463 (38.5)
**Demography** Female	3849 (48.8)	586 (48.7)
Age (years) 0-4 5-14 15-29 30-44 45-59 >=60	1542 (19.5) 418 (5.3) 805 (10.2) 1460 (18.5) 1551 (19.7) 2110 (26.8)	94 (7.8) 68 (5.6) 142 11.8) 352 (29.3) 338 (28.1) 209 (17.4)
Inpatient severity Deaths	529 (6.7)	196 (16.3)

**Daily epidemic curves of all SARI hospitalizations (top) and deaths (bottom) by dates of symptoms onset in northern, central, and southeastern states of Mexico, 01-December 2013 to 25-January 2014.**
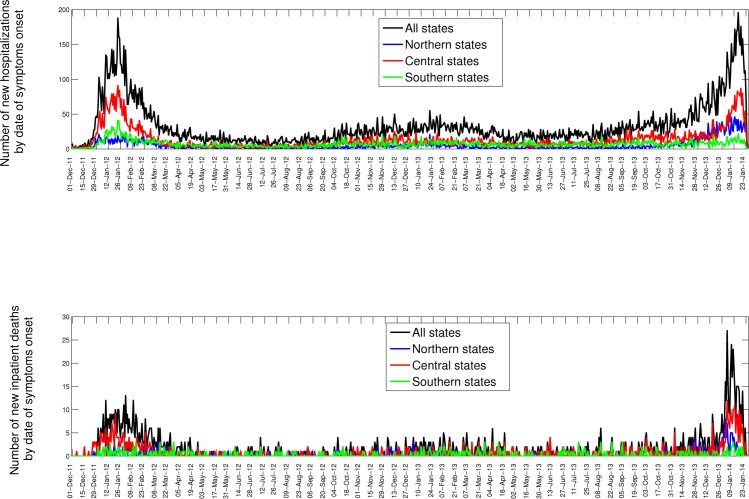

**Daily number of influenza tests among SARI hospitalizations and laboratory-confirmed influenza hospitalizations by dates of symptoms onset spanning 01-December 2011 to 25-January 2014.in the 32 Mexican states according to influenza subtype.**
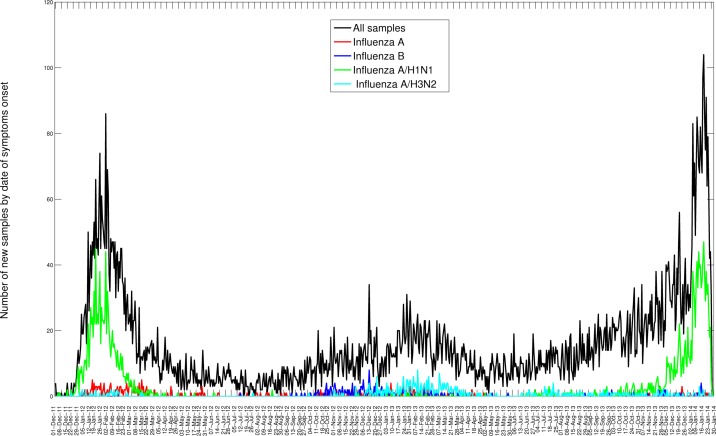

**Severity of disease**

Between October 2013 and January 2014, 529 inpatient-SARI deaths were reported among 7,886 SARI hospitalizations, with a preliminary case fatality rate of 6.7% (95% CI: 6.1, 7.3). The preliminary estimate of hospital CFR for laboratory-confirmed A/H1N1 was 16.3% (95% CI: 14.2, 18.4) over the same period (1203 inpatients and 196 deaths). The A/H1N1-specific CFR estimate for the 2013-2014 epidemic was similar to that of earlier A/H1N1 seasons in Mexico, including the 2011-12 epidemic (13.8% (95% CI: 11.7, 15.9)) and the 2009 pandemic (16.1% (95% CI: 15.0, 17.2)). Hospital CFRs were not significantly different across geographic regions (Chi-square test, P=0.34).

**Age patterns**

Overall the majority of laboratory-confirmed influenza inpatients during Oct 2013 - Jan 2014 were among persons aged 30-59 years (57.4%) followed by seniors >=60 years (17.4%). Severity increased with older age, with a hospital CFR of 22.5% (95% CI: 16.7, 28.3) in seniors. Laboratory testing practices were similar between age groups, with 49% of SARI hospitalizations tested for influenza in persons <30 yrs , 54% among people 30-59 yrs and 48% among older individuals.

Comparison of the age-specific A/H1N1 hospitalization and death rates between the 2013-2014 season and previous A/H1N1 epidemic in 2011-2012 reveals an increasing burden among adults (>=30 yrs), particularly among middle-aged individuals (Figure 3). The shift is seen both in absolute and relative terms. Specifically, we note a significantly higher proportion of individuals aged 30-59 yrs hospitalized with laboratory-confirmed A/H1N1 in 2013-14, relative to the 2011-12 A/H1N1 epidemic, as well as a significant decrease in the proportion hospitalizations among younger persons (Chi-square test, df=5, P<0.0001).

We found a similar change in the age distribution of A/H1N1 inpatient deaths in 2013-14 compared to the 2011-12 A/H1N1 influenza epidemic. Specifically, 67.4% of deaths occurred among persons 30-59 years of age in the ongoing 2013-14 epidemic period whereas only 50% in the 2011-12 A/H1N1 epidemic. Similarly to the age shift in hospitalization data, the proportion of A/H1N1 inpatient deaths among individuals aged 0-29 declined, relative to the 2011-12 A/H1N1 epidemic (Chi-square test, df=5, P=0.002)

**Age-specific A/H1N1 influenza hospitalization rates (left) and A/H1N1 inpatient death rates (right) for the ongoing A/H1N1 influenza epidemic (01-Oct 2013 to 31-Jan 2014) compared to the preceding 2011-12 A/H1N1 epidemic and those of the entire 2009 A/H1N1 pandemic period (01-Apr 2009 to 31-Mar 2010).**
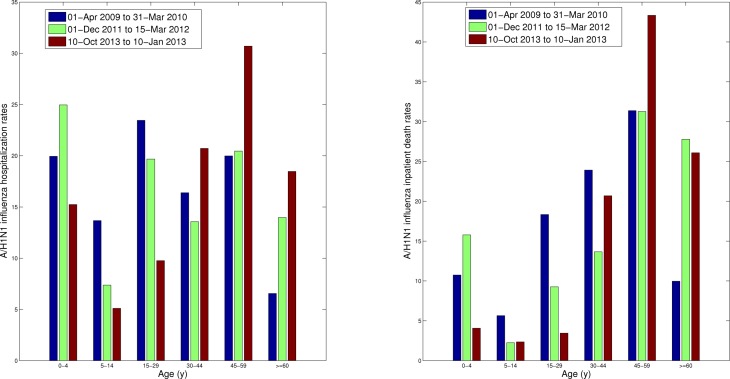

**Estimates of the reproduction number**

Assuming a mean generation interval of 3 (4) days, the mean R was estimated to be 1.3 (1.4) during the monotonically ascending period 04-Dec 2013 to 08-Jan 2014, based on daily laboratory-confirmed A/H1N1 case series in central Mexican states. As a sensitivity analysis we also estimated R using a slightly shorter ascending phase and obtained a similar estimate at 1.2 (1.3) (Figure 4). Our R estimate was slightly higher than that estimated for the 2011-12 A/H1N1 season, with R at 1.2 (1.3).

**Model fits (solid line) obtained after fitting an exponential curve to the growth phase of the initial epidemic phase of the 2013-14 A/H1N1 epidemic to estimate the initial growth rate based on the case series of laboratory-confirmed A/H1N1 SARI A/H1N1 hospitalizations (left) and using a slightly shorter ascending phase as a sensitivity analysis (right).**
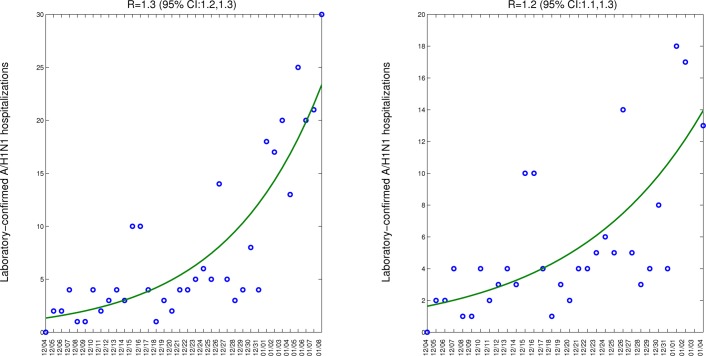

## DISCUSSION

We have characterized the epidemiology of a recrudescent wave of pandemic A/H1N1 influenza activity in Mexico during October 2013-January 2014, based on individual-level laboratory-confirmed influenza hospitalizations and deaths, and compared with the well-described epidemiology of earlier A/H1N1 seasons 1 ^,^ 3. Our data show that the level of hospitalizations in 2013-2014 has exceeded that of the last A/H1N1 epidemic in 2011-12. We have also documented a significant shift in absolute and relative severe disease burden from young individuals (<30 yrs) to middle-aged adults 30-59 years, relative to the preceding A/H1N1 epidemic.

The observed gradual change in age distribution of hospitalization and deaths in the post 2009 pandemic period is reminiscent of the influenza seasons following the 1918 influenza pandemic 6 ^,^ 18 ^,^ 19 and the 1968 pandemic 20. A quantitative analysis of excess mortality in the decade following the 1918 influenza pandemic found that the age distribution of influenza-related mortality returned to pre-pandemic mortality levels a few years after the initial pandemic waves as a result of emerging drift variants^6^^,^ 21. Hence the age shift seen in the 2011-12 and 2013-14 A/H1N1 epidemics could signal a build up of immunity among younger populations, which has implications for influenza prevention and mitigation strategies.

It is now well recognized that during the first year of circulation of the 2009 A/H1N1 influenza pandemic virus, seniors over 60 years enjoyed protection from influenza-related morbidity and mortality. This phenomenon of “senior sparing” in age cohorts born prior to the 1957 pandemic is consistent with the protective effect of first exposure to antigenically-related A/H1N1 viruses in childhood, which aligns with antigen recycling and original antigenic sin hypotheses^22^^,^ 23 ^,^ 24 ^,^ 25 ^,^ 26. A high fraction of the Mexican population is now presumably protected against the 2009 A/H1N1 influenza virus through natural exposure since 2009 (children and young adults), prior immunity (seniors) 27 and by pandemic vaccines. In parallel, waning of immunity is also expected in individuals that are not periodically re-vaccinated. To the best of our knowledge, no serological studies were conducted prior to this epidemic or are currently being carried out in Mexico.

For the first time since the emergence of the 2009 pandemic virus, the absolute risk of hospitalization is greater among individuals >=30 yrs compared to that of individuals <30 yrs in Mexico (Figure 3). The declining rates of severe cases in younger age groups is most consistent with the build-up of immunity in these age groups. In contrast, seniors still experience relative protection against A/H1N1 hospitalization and death compared to persons aged 30-59 yrs in the current epidemic, indicating residual immunity in this population. Residual protection among seniors is consistent with a lack antigenic drift in A/H1N1 viruses circulating in Mexico or elsewhere in winter 2013-14. Overall the age distribution of A/H1N1 is gradually progressing to an older adult age risk profile that characterizes seasonal influenza but is not yet that of a typical inter-pandemic season, with 90% of deaths occuring in seniors. In the longer term, we expect the pandemic A/H1N1 virus to drift genetically to escape mounting population immunity – perhaps with the result that seniors are less protected 6.

We estimated a slightly higher reproduction number for the ongoing A/H1N1 epidemic (R = 1.3 (95% CI: 1.2-1.3) than for the 2011-2012 A/H1N1 epidemic (R ~1.2 95% CI: 1.1-1.2). We note that even a slight difference in R, similar to that reported here, results in a substantial difference in final epidemic size and in the size of interventions required to stop transmission. The slightly higher R measured for the ongoing epidemic could be explained by differences in population immunity through vaccination or natural exposure. Since transmission of the A/H1N1 influenza virus was sporadic in the previous winter of 2012-2013 in Mexico, population immunity may have slightly waned. Further, the early timing of onset of the on-going 2013-2014 season (early October 2013, Figure 2) could have prevented the population from getting immunized and reaching protective antibody levels by the time transmission started. Of note, influenza vaccination coverage among the IMSS population has increased in the last three seasons and mid-December estimates were 8.7% (2011), 11% (2012) and 14% (2013). Influenza vaccination through IMSS is focused primarily on high-risk groups including infants, pregnant women, health care workers and seniors. In particular, among seniors >=60 yrs. the influenza vaccination coverage was ~16% in 2011-12 and 2012-13 and 29% in 2013-2014. Finally, we cannot rule out that reporting or measurement issues could account for the observed differences in estimated Rs.

It is also worth comparing the transmission dynamics of the 2013-2014 season with that of the 2009 pandemic. Our R estimate for the ongoing epidemic is lower to that of the spring (R~1.8-2.1) and summer (R~1.6-1.9) pandemic waves in 2009 in Mexico, but similar to estimates for the fall (3rd) 2009 wave (R~ 1.2-1.3) 1 . These declining trends are consistent with a gradual build up of immunity in the first few months and years of A/H1N1 circulation. Further, the gradual shift of A/H1N1 infection towards adults, who transmit less than children due to behavioral and biological differences, may contribute to the post-pandemic decline in R.

Most countries have experienced several waves of A/H1N1 pandemic activity by now. The Mexican 2013-14 outbreak coincides with a highly-publicized recrudescence of A/H1N1 activity in North America, associated with an increase in young adults hospitalizations and deaths in Canada and the US 28. Although there is no serological information specific to Mexico, Canadian surveys indicate low levels of immune protection in young and middle-aged adults prior to the 2013-14 winter, in contrast to children, potentially explaining the recrudescence of young adult cases observed in 2013-14 in the region 28. The epidemiology of the 2013-14 Mexican outbreak also echoes the increased severity and transmissibility of the 3^rd^ pandemic wave in the UK in 2011-12 29. Gradual build-up of A/H1N1-specific humoral immunity in various age groups likely explains some of the differences reported between countries and successive pandemic waves. However the increased propensity of young and middle aged adults to be infected in later waves of the pandemic, rather than during the initial outbreak, remains intriguing, and could reflect the dynamics of contact rates 30 or non-specific T-cell mediated immunity 31.

In summary our findings indicate a changing age distribution of laboratory-confirmed A/H1N1 influenza hospitalizations and deaths in winter 2013-14, relative to the preceding A/H1N1 seasons in 2011-2012 and 2009-10. The proportion of hospitalizations and deaths is increasing particularly among middle aged adults. In contrast, rates of A/H1N1 hospitalizations and deaths are declining significantly among children, consistent with a gradual build up of immunity. This epidemiological shift is reminiscent of outbreaks in historical post-pandemic periods and consistent with on-going influenza outbreaks elsewhere in North America. As this epidemic is still underway, it is too early to determine whether its cumulative mortality impact will be more susbtantial than the previous waves, similarly to the 1889 pandemic 10 32. A systematic multinational comparison of the epidemiology of pandemic and post-pandemic waves would be useful to shed light on the transmission dynamics and build up of immunity to pandemic viruses, and inform long-term control strategies.

## Competing interests

The authors declare no relevant competing interests.
